# Challenges in Resource Utilization for Caregivers of Persons With Dementia: A qualitative Study

**DOI:** 10.1093/geroni/igab046.2249

**Published:** 2021-12-17

**Authors:** Robert Turner, Jen Weaver, Eric Owens, Meredith Boe, Jessica Bride, Maritza Dowling, Christina Prather, Melinda Power

**Affiliations:** 1 The George Washington University, Washington, District of Columbia, United States; 2 George Washington University - School of Nursing, Washington, District of Columbia, United States; 3 The GW Medical Faculty Associates, Washington, District of Columbia, United States

## Abstract

This study highlights primary caregivers’ experiences with health department policies designed to support people with cognitive impairment/Alzheimer’s Disease and Related Dementias (ADRD). Caregivers were defined as individuals aged 45-85 that provide at least 10 hours of unpaid care. Five, 90-minute focus groups were conducted virtually with 24 caregivers of individuals with cognitive impairment/ADRD. Transcripts were analyzed thematically. Caregivers were primarily Black females (75%) with at least a high school education (42%). Care recipients were likely to be community-dwelling parents (71%), with moderate or advanced (79%) dementia. Caregivers described challenges with accessing resources intended for care recipients, especially as cognitive impairment worsened. Caregivers reported providing care 24/7 as traumatizing. Home-based personal aides and companionship services did not reduce this burden. COVID-19 impacted caregivers and care recipient’s access to resources increasing burden. Policies need to be flexible for ever-changing needs of individuals with ADRD and support the overall well-being of the caregivers.

